# Meta-analysis of expression signatures of muscle atrophy: gene interaction networks in early and late stages

**DOI:** 10.1186/1471-2164-9-630

**Published:** 2008-12-23

**Authors:** Enrica Calura, Stefano Cagnin, Anna Raffaello, Paolo Laveder, Gerolamo Lanfranchi, Chiara Romualdi

**Affiliations:** 1 Department of Biology, University of Ferrara, Via L. Borsari 46 I-44100 Ferrara, Italy; 2 CRIBI Biotechnology Centre and Department of Biology, University of Padova, via U. Bassi 58/B 30121 Padova, Italy

## Abstract

**Background:**

Skeletal muscle mass can be markedly reduced through a process called atrophy, as a consequence of many diseases or critical physiological and environmental situations. Atrophy is characterised by loss of contractile proteins and reduction of fiber volume. Although in the last decade the molecular aspects underlying muscle atrophy have received increased attention, the fine mechanisms controlling muscle degeneration are still incomplete. In this study we applied meta-analysis on gene expression signatures pertaining to different types of muscle atrophy for the identification of novel key regulatory signals implicated in these degenerative processes.

**Results:**

We found a general down-regulation of genes involved in energy production and carbohydrate metabolism and up-regulation of genes for protein degradation and catabolism. Six functional pathways occupy central positions in the molecular network obtained by the integration of atrophy transcriptome and molecular interaction data. They are TGF-β pathway, apoptosis, membrane trafficking/cytoskeleton organization, NFKB pathways, inflammation and reorganization of the extracellular matrix. Protein degradation pathway is evident only in the network specific for muscle short-term response to atrophy. TGF-β pathway plays a central role with proteins SMAD3/4, MYC, MAX and CDKN1A in the general network, and JUN, MYC, GNB2L1/RACK1 in the short-term muscle response network.

**Conclusion:**

Our study offers a general overview of the molecular pathways and cellular processes regulating the establishment and maintenance of atrophic state in skeletal muscle, showing also how the different pathways are interconnected. This analysis identifies novel key factors that could be further investigated as potential targets for the development of therapeutic treatments. We suggest that the transcription factors SMAD3/4, GNB2L1/RACK1, MYC, MAX and JUN, whose functions have been extensively studied in tumours but only marginally in muscle, appear instead to play important roles in regulating muscle response to atrophy.

## Background

Atrophy is a complex modification occurring in skeletal muscles as a result of a variety of causes such as damages to neural connections, disuse or unloading, fasting and also as a consequence of many diseases including diabetes, sepsis, acidosis or cancer. The variety of conditions inducing atrophy implies different molecular triggers and signalling pathways for muscle wasting. However, regardless of the stirring event, skeletal muscle atrophy is generally characterized by a decrease in protein content, fiber diameter, force production, and fatigue resistance.

The dynamic regulation of skeletal muscle mass depends on the balance between overall rates of protein synthesis and degradation. It is now established that these two biochemical processes appear to be coordinated by complex signalling networks. During hypertrophy, the rate of synthesis of muscle contractile proteins is much higher than the rate of degradation that results in an increase of the size of the existing muscle fibers. On the contrary, enhanced protein breakdown is the primary cause of the rapid loss of muscle proteins that occurs during atrophy [1-3].

Significant advancements have been recently made in the understanding of the signalling pathways mediating skeletal muscle atrophy and its opposite process of hypertrophy [4-9]. It has become clear that the activity or inactivity of the IGF-1/Insulin/Akt/FoxO pathway determines whether a muscle will increase protein synthesis and growth (hypertrophy), or undergo protein breakdown and atrophy. In particular, IGF-1 stimulation induces hypertrophy of skeletal muscle by stimulating the phosphatidylinositol 3-kinase (PI3K)-Akt pathway, resulting in the downstream activation of proteins required for protein synthesis [10,11]. Downstream of PI3K-Akt signal, IGF-1 activates also mTOR and p70S6K. However, mTOR can be activated directly by amino acids, causing a subsequent stimulation of p70S6K activity [12,13]. Thus, mTOR seems to have a central role in integrating a variety of growth signals, from simple nutritional stimulation to activation by protein growth factors, resulting in protein synthesis. Akt activates mTOR by phosphorylation [14], and both Akt and mTOR phosphorylation are increased during muscle hypertrophy [15]. Conversely, when the activity of the IGF-1/Akt/FoxO pathway decreases, the transcription factors FoxO1 and 3 are activated and the two muscle specific E3 ubiquitin ligases atrogin-1 (or MAFbx, muscle atrophy F-box) and MuRF-1 (muscle ring finger 1) are induced [16,17]. These proteins have been identified by genomic experiments designed to uncover new markers of the atrophy process [18,19] and their expression is increased significantly in several types of muscle atrophy, demonstrating the predominant role of the ubiquitin-proteasome pathway during the progression of muscle wasting [20].

The NFKB signalling cascade also plays an important role in the control of muscle degradation. First hints on involvement of NFKB in muscle wasting came from the up-regulation of this gene during disuse atrophy [21] and sepsis [22]. Additionally, experiments in cultured myotubes demonstrated that the block of this transcription factor by overexpression of a mutant form of I-kBα, that is insensitive to degradation by the proteasome, inhibits protein loss induced by tumor necrosis factor-α (TNF-α) [23].

Not only the IGF-1/PI3K/Akt/FoxO and the NFKB signalling cascades are involved in the control of muscle mass upon skeletal muscle atrophy. Recent studies in cultured myotubes, mouse models and natural mutations demonstrated that also myostatin is a potent regulator of skeletal muscle mass [24-28]. The mechanism by which myostatin inactivation leads to muscle growth is still controversial. Recently, it was proposed that the myostatin signalling pathway could be linked to the IGF-1/PI3K/Akt pathway. McFarlane and co-workers showed in cultured myotubes and in mouse skeletal muscle that treatment with myostatin was associated with a reduction of fiber size, and with induction of the muscle-specific E3 ubiquitin ligases atrogin-1 and MuRF-1 [29]. This study showed that the atrophic effects observed were mediated by dephosphorylation and inhibition of Akt and the consequent activation of FoxO1.

Although in the last decade, with the application of genomic technologies such as global gene expression profiling, the molecular networks underlying several types of atrophy have been studied in deeper details, the fine mechanisms that control muscle wasting and loss of functional capacity are still incomplete. The different types of atrophy may involve the coordinated action of a wide number of genes organised in complex networks and the outcome of individual expression studies is insufficient for the complete comprehension of such composite state. Instead, it would be meaningful for this purpose to undertake approaches aimed to combine and integrate data from the various studies that, at different level of resolution, have been applied to muscle atrophy. The outcome of a data integration approach for complex phenomena like muscle atrophy may not only be used for the confirmation and strengthening of results of single studies, but also for the completion of common molecular pathways, the definition of pivotal gene players and hopefully the individuation of novel players and pathways. The integrative analysis of multiple gene expression datasets concerning a common biological problem, called "meta-analysis", has already demonstrated the capability of retrieving much more relevant information than single experiment datasets [30,31].

In this paper we present the results of a comprehensive meta-analysis performed on publicly available gene expression datasets pertaining to different types of muscle atrophy caused by aging [32], fasting [20], unloading [33], denervation [5,34], and by a number of diseased states like uremia, diabetes and cancer cachexia [4], in human, mouse and rat models. Our study was designed to pursue multiple goals such as the identification of novel possible key regulatory signals implicated in muscle wasting caused by atrophic states and the definition in the signalling pathways of similarities shared by different atrophic states or across evolutionary related mammalian species.

As expected, we found a significant enrichment associated to up-regulation of biological processes related to catabolism and protein degradation, and a significant enrichment with down-regulation of processes related to energy production (ATP production, oxidoreductase activity, CREB cycle, glycolysis) and muscle development. The comparison of enriched functional categories separates atrophies caused by long-term stimuli from those caused by short-term stimuli. Furthermore, we studied the enrichment of specific transcription factor (TF) binding sites in the genes relevant for atrophy as revealed by the meta-analysis, obtaining clues for important roles that should be ascribed in the atrophy processes to TF such as SP1, MAX and EEF1D/deltaEF1. Interestingly, some TF genes that target these enriched sequences appear to be also differentially expressed in most of the atrophy datasets. The combination of these two results strength the output of meta-analysis and then allows the reconstruction of specific regulatory pathways.

The integration of transcriptional signatures derived by single studies with molecular interaction data has allowed the reconstruction of a complex molecular network that includes genes deregulated in at least one type of atrophy. Focusing on the hub transcripts that are deregulated in at least three of these studies, and selecting only highly connected nodes and their nearest neighbours edges, we zoomed into the networks identifying specific pathways commonly involved in the atrophic process. The TGF-β pathway seems to be the core of the network with SMAD3/4, MYC, MAX, SP1, CDKN1A/B proteins involved in regulating cell cycle and differentiation of many cell types included skeletal muscle cells. Additional pathways representative of cell reaction to cycle arrest were identified as separate areas of the network: NFKB pathway, apoptosis, membrane trafficking, cytoskeleton organization and inflammation.

We applied the same approach separately to muscle expression datasets obtained at short distance from the initiation of atrophic process, revealing that the up-regulation of genes involved in proteolytic and catabolic processes characterizes the early muscle response to atrophic stimuli. The meta-analysis of expression signatures of muscles at 14 or more days from atrophy initiation shows that this early response became somehow balanced. Hub genes occupying a central role in the molecular networks of atrophy short-term response overlap only partially those found with the general meta-analysis. In particular, MYC and MAX genes are found by both analysis, whereas SMAD3/4 proteins seem to be replaced by JUN and GNB2L1/RACK1 in the short-term signature.

## Results and discussion

Skeletal muscle accounts for almost 40% of adult human body mass and is composed by a differentiated and specialized tissue characterized however by a high rate of plasticity to adapt to physiological changes. As a consequence of many diseases or critical physiological and environmental conditions, skeletal muscle mass can be markedly reduced, through a process generally called atrophy. This process is characterised by depletion of contractile proteins and reduction of muscle fiber volume. Although muscle atrophy can be induced by a variety of very diverse stimuli, there are a number of unexpected similarities among the intracellular responses that mediate the atrophic processes. The aims of our study were *i*) the definition of genes and pathways specifically involved in one or few types of atrophies in order to gain new insights in the mechanism controlling atrophy; *ii*) the comparison of molecular pathways underlying different types of atrophy to identify shared core molecular mechanisms leading to muscle wasting. A detailed description of datasets included in the meta-analysis is available in Table 1.

**Table 1:** Atrophy gene expression datasets used for the meta-analysis

**Authors**	**Journal**	**Year**	**Atrophy**	**Platform**	**Organism**	**Tot. Probes**	**Muscle Type**	**Experimental Design**
Kostrominova et al.	*Physiol. Genomics*	2005	Denervation	Membrane arrays	*R. Norvegicus*	1,176	Extensor Digitorum Longus	Case vs Control

Raffaello et al.	*Physiol. Genomics*	2006	Denervation	cDNA arrays	*M. Musculus*	2,061	Tibialis Anterioris	Time course

Sacheck et al.	*FASEB*	2007	Denervation Spinal Cord	cDNA array	*R. Norvegicus*	8,734	Gastrocnemius	Case vs Control

Welle et al.	*Physiol. Genomics*	2003	Aging	Affymetrix HG-U133A/B	*H. Sapiens*	44,928	Vastus Lateralis	Case vs Control

Stevenson et al.	*J Physiol*.	2003	Unloading	Affymetrix U34A	*R. Norvegicus*	8,799	Soleus	Time course

Jagoe et al.	*FASEB*	2002	Fasting	cDNA array	*M. Musculus*	8,734	Gastrocnemius	Case vs Control

Lecker et al.	*FASEB*	2004	Systemic wasting states	cDNA array	*R. Norvegicus*	8,734	Gastrocnemius	Case vs Control

### Similarities of functional category enrichment among different atrophies

**Figure 1:**
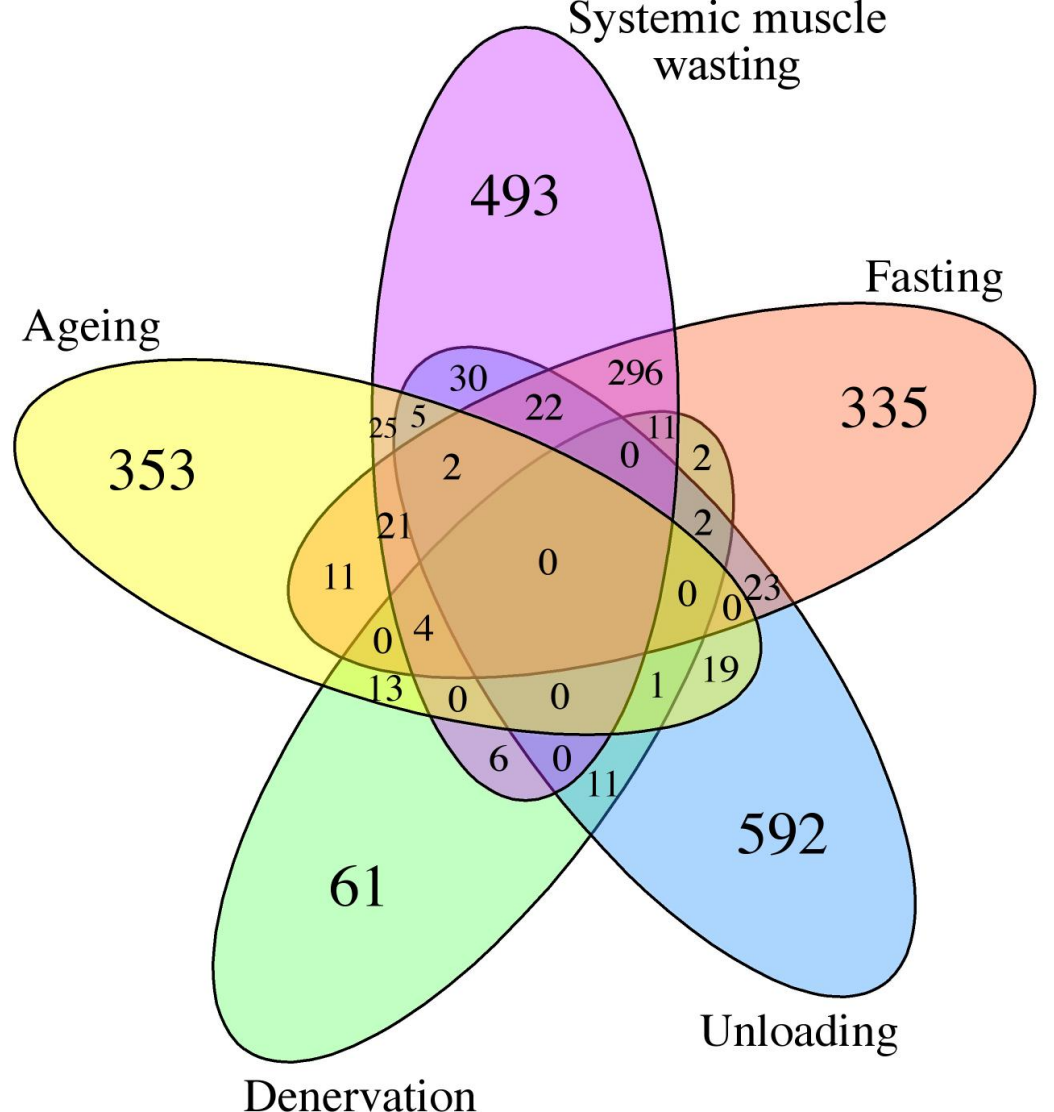
**Numbers of differentially expressed genes in the atrophy datasets analyzed in this study and overlaps across each of them**.

Initially, the identification of differentially expressed genes has been performed separately for each dataset. The numbers of differentially expressed genes detected specifically in each independent dataset and the overlap between them are reported respectively in Table 2 and Figure 1.

**Table 2:** Number of differentially expressed genes of the datasets included in the meta-analysis.

**Authors**	**Up-regulated**	**Down-regulated**	**Tot. N. Probes**
Kostrominova et al.	(75) 6.4%	(29) 2.5%	1,176

Raffaello et al.	(370) 18%	(404) 20%	2,061

Sacheck et al.	(923) 10%	(545) 6%	9,130

Welle et al.	(194) 1.6%	(264) 2.2%	11,991

Stevenson et al.	(316) 7.8%	(436) 11%	4,029

Jagoe et al.	(413) 4.7%	(412) 4.7%	8,734

Lecker et al.	(496) 5.7%	(550) 6.3%	8,734

Percentage values are calculated with respect to the total number of probes in the microarray platform used in each study.

As expected, there are no commonly deregulated genes resulting from the intersection of all five atrophy profiles; this is probably due to the experimental differences between studies included in the work (stimuli inducing atrophy, microarray platforms, muscle types, and organisms, see Table 1). With the exception of fasting and systemic muscle wasting, about 60–80% of the genes in each list seem to be stimulus-specific, while the remaining 20–40% seems to be shared by different groups of atrophies (Figure 1). The groups of differentially expressed genes in fasting and systemic muscle wasting show a striking overlapping percentage. Both groups of genes derived from experiments performed with the same microarray platform and the same muscle type, while all the other groups derived from experiments obtained through the combination of different platforms and muscle types (see Table 1). We are confident that the larger overlapping percentage with respect to the other comparisons could be partially due to these similarities.

Recently, Hosack et al. [35] have shown that different selection and comparison methodologies of expression data can result in gene lists that differ in quality and quantity of genes, but they also show that in spite of this variation the top five most represented biological categories in which differentially expressed genes are classified remain constant. This means that meta-analysis approaches give consistent results when comparing functional classes (e.g. specific cellular function or metabolic pathway) with respect to genes.

In the light of these findings, GO functional category enrichment has been performed for each list of deregulated genes, identifying those functional categories (biological process, molecular function, cellular component and metabolic pathways) commonly shared by the studies. Table 3 and 4 report the enriched categories shared by the datasets. As expected, we found a general significant enrichment of functional categories related to catabolism processes (proteasome pathways, autophagy, catabolism), in which are classified mostly over expressed genes, and of functional classes related to energy production with carbohydrate metabolism (oxidoreductase activity, reductive carboxylate cycle, response to hypoxia, oxidative phosphorylation, nitrogen metabolism) in which instead are classified mostly down regulated genes. In atrophy, in fact, the rate of degradation of contractile proteins becomes greater than the rate of replacement, modifying the balance requested for the maintenance of skeletal muscle mass. In addition, we found also over represented the functional categories of muscle contraction and development linked to muscle wasting state, and of insulin signalling pathway that have been extensively associated to muscle hypertrophy and atrophy.

**Table 3:** Common KEGG pathways enriched in the different expression datasets.

	**K**	**L**	**J**	**R**	**W**	**St**	**Sc**	**Tot**	**Expr**.
***Array platform type***	*Membrane*	*cDNA*	*cDNA*	*cDNA*	*Affy*	*Affy*	*cDNA*		
***Muscle fibre composition***	*fast*	*Mixed*	*mixed*	*fast*	*mixed*	*slow*	*mixed*		
***Organism***	*RN*	*RN*	*MM*	*MM*	*HS*	*RN*	*RN*		
**Energy Metabolism**									
Nitrogen metabolism		X	X	X	X	X	X	**6**	**-**
Carbon fixation		X	X	X	X	X	X	**6**	**-**
Crebb Cycle		X	X	X	X		X	**5**	**-**
Oxidative phosphorylation		X	X			X	X	**4**	**-**
**Carbohydrate Metabolism**									
Glycolysis/Gluconeogenesis		X	X	X		X	X	**5**	**-**
Pyruvate metabolism		X	X		X	X	X	**5**	
Citrate cycle (TCA cycle)		X	X	X	X		X	**5**	
Pentose phosphate pathway		X		X		X	X	**4**	**-**
Glyoxylate and dicarboxylate metabolism		X	X		X		X	**4**	
Butanoate metabolism		X	X			X	X	**4**	
**Amino Acid Metabolism**									
Cysteine metabolism	X	X		X		X	X	**5**	
Phenylalanine, tyrosine and tryptophan biosynthesi		X	X	X		X	X	**5**	
Valine, leucine and isoleucine degradation		X	X		X	X	X	**5**	
Lysine degradation		X	X			X	X	**4**	
Phenylalanine metabolism		X	X	X			X	**4**	
Methionine metabolism	X	X	X		X			**4**	
Urea cycle and metabolism of amino groups		X			X	X	X	**4**	
**Neurodegenerative Diseases**									
Neurodegenerative Disorders		X	X			X	X	**4**	
Parkinson's disease		X	X	X			X	**4**	
Alzheimer's disease	X		X			X	X	**4**	
**Other**									
Glutathione metabolism	X	X		X		X	X	**5**	
Proteasome		X	X	X	X		X	**5**	**+**
Ribosome	X	X		X	X		X	**5**	
Focal adhesion	X	X	X				X	**4**	
Insulin signalling pathway		X	X	X			X	**4**	
Complement and coagulation cascades		X		X	X	X		**4**	

K: Kostrominova et al.; L: Lecker et al.; J: Jagoe at al.; R: Raffaello et al.; W: Welle et al.; St: Steavenson et al.; Sc: Sacheck et al. X indicates if the category is significantly enriched in the corresponding study (FDR < = 0.1). In the organism row: RN is *Rattus Norvegicus*, MM *Mus Musculus*, HS *Homo Sapiens*. The Expr column indicates the up (+) or down (-) regulation of genes belonging to each class. When the sign is not reported it means that gene belonging to that class are characterised by a mixed expression regulation.

**Table 4:** Common GO categories enriched in the different expression datasets.

	**K**	**L**	**J**	**R**	**W**	**St**	**Sc**	**Tot**
***Array platform type***	*Membrane*	*cDNA*	*cDNA*	*cDNA*	*Affy*	*Affy*	*cDNA*	
***Muscle fibre composition***	*Fast*	*mixed*	*mixed*	*fast*	*mixed*	*slow*	*mixed*	
***Organism***	*RN*	*RN*	*MM*	*MM*	*HS*	*RN*	*RN*	
**Biological Process**								
Response to radiation	X	X		X	X		X	**5**
Response to hypoxia		X	X	X	X		X	**5**
Glucose homeostasis		X	X	X	X		X	**5**
Cellular metabolism	X	X	X	X				**4**
Ion homeostasis		X	X			X	X	**4**
Macromolecule metabolism	X	X	X	X				**4**
Muscle development	X	X	X	X				**4**
Negative regulation of enzyme activity	X	X	X	X				**4**
Regulation of cell growth	X	X	X	X				**4**
Transport			X	X		X	X	**4**
Biomineral formation	X	X	X	X				**4**
Biosynthesis	X	X	X	X				**4**
Muscle contraction	X	X	X	X				**4**
Osteoblast differentiation	X	X	X	X				**4**
Regulation of coagulation			X	X		X	X	**4**
Response to chemical stimulus	X	X	X	X				**4**
Autophagy		X	X	X				**3**
Striated muscle contraction					X	X	X	**3**
Adult behaviour		X	X				X	**3**
Catabolism		X	X	X				**3**
								
**Molecular Function**								
Coenzyme binding		X	X	X	X	X	X	**6**
Transferase activity, transferring alkyl or aryl (other than methyl) groups	X	X	X	X		X	X	**6**
Oxidoreductase activity		X	X	X	X	X	X	**6**
Cytoskeletal protein binding	X	X		X	X	X	X	**6**
Growth factor binding	X	X	X			X	X	**5**
Primary active transporter activity	X	X	X	X			X	**5**
Carbon-oxygen lyase activity		X		X	X	X	X	**5**
Ligand-dependent nuclear receptor activity		X	X		X	X	X	**5**
Phospholipid binding		X	X	X	X		X	**5**
Translation factor activity, nucleic acid binding	X	X	X	X			X	**5**
Cation transporter activity		X	X	X			X	**4**
Peptidase activity	X	X				X	X	**4**
Hydrolase activity, acting on glycosyl bonds	X	X			X		X	**4**
Metal ion transporter activity		X	X	X		X		**4**
Electron carrier activity			X	X		X	X	**4**
Intramolecular transferase activity		X		X		X	X	**4**
Iron-sulfur cluster binding			X	X	X		X	**4**
L-ascorbic acid binding		X	X	X			X	**4**
Ligase activity, forming carbon-oxygen bonds		X	X		X		X	**4**
Diacylglycerol binding	X	X	X	X				**4**
Fatty acid binding			X	X		X	X	**4**
GTP-dependent protein binding			X	X	X	X		**4**
Transcription factor binding		X	X	X			X	**4**
Transferase activity, transferring phosphorus-containing groups		X	X			X	X	**4**
								
**Cellular Component**								
Cytoplasm	X	X	X	X				**4**
Glycerol-3-phosphate dehydrogenase complex		X	X	X			X	**4**
Intracellular non-membrane-bound organelle	X	X	X	X				**4**
Mitochondrial envelope	X	X	X	X				**4**
Organelle inner membrane	X	X	X	X				**4**
Ribonucleoprotein complex	X	X	X	X				**4**
Ribosome	X	X	X	X				**4**
Insulin-like growth factor binding protein complex		X	X	X				**3**
Intracellular membrane-bound organelle		X	X	X				**3**
Intracellular organelle		X	X	X				**3**
Organelle membrane		X	X	X				**3**
Soluble fraction		X	X	X				**3**
Intracellular					X	X	X	**3**
Organelle envelope		X	X	X				**3**
Proteasome complex (sensu Eukaryota)		X	X	X				**3**
Basement membrane	X		X	X				**3**
Cytoskeleton	X	X		X				**3**

Symbols are as in the Table 3. X indicates if the category is significantly enriched in the corresponding study (FDR < = 0.1).

**Figure 2:**
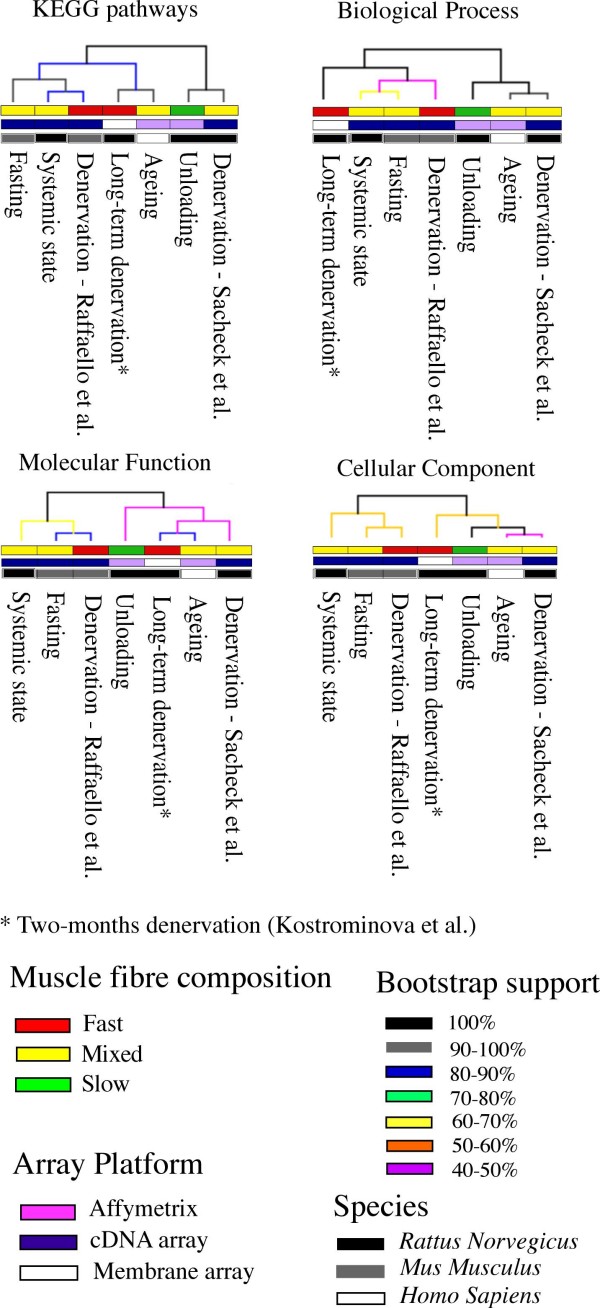
**Dendrograms showing the GO category enrichment similarities among atrophy gene expression datasets**. GO functional categories (biological process, molecular function and cellular component) and KEGG pathways have been analysed separately. Muscle fibre compositions, microarray platform type and organism have been indicated to evaluate whether these features could influence the structure of dendrograms.

To evaluate the degree of functional similarity among datasets, we used functional enrichment *p-values *as a measure to obtain a similarity matrix. The functional categories listed in Table 3 and 4 are the rows of the matrix and the seven atrophy studies are indicated in the columns (Table 1); the cells of the matrix contain the corresponding enrichment *p-value*. Cluster analyses have been performed, through TMEV tool [36], using GO and KEGG functional matrices and Figure 2 shows the resulting dendrograms. Different p-value transformations have been used to test the dendrogram robustness: dendrograms on datasets (columns) did not show changes in the whole structure but only on the bootstrap support (however, the first separation of datasets in two broad classes was always characterised by 100% bootstrap support). The clustering of expression datasets seems to be independent from muscle type or microarray platforms, but rather influenced from the type of stimulus inducing atrophy. Except for some little differences, dendrograms of Figure 2 underline the presence of two broad groups with similar profiles of over represented functional categories: *i*) unloading [33], ageing [32], long-term denervation response [37], denervation and spinal cord isolation [5] and *ii*) fasting [20], systemic muscle wasting [4] and short-term response to denervation [34]. It should be noted that the gene expression studies of Sacheck et al. [5] and Lecker et al. [4] have been performed using respectively rat muscle mRNAs hybridised to a human cDNA microarray and rat muscle mRNAs hybridised to a mouse cDNA microarray. A possible problem of the use of heterologous microarray platforms and hybridizations is probe-target sequence mismatches. In the presence of such sequence mismatches, relative hybridization intensities will reflect both differences in transcript abundance (the object of interest), as well as differences in hybridization kinetics. Gilad et al. [38] showed that sequence divergence between probes in the platform and test RNA can have substantial effects on estimates of expression levels, even for evolutionary close species such as human and chimpanzee. Thus, Sacheck et al. [5] and Lecker et al. [4] results should be used carefully even if the use of group of genes such as GO/KEGG categories rather than single genes should reduce possible biases. Nevertheless, our analysis shows that expression datasets are separated in two groups of similarity, according to the nature of the stimulus inducing atrophy: long-term versus short-term. In this case the use of short and long term is referred to the time required for the development of atrophy condition rather to the time points used for expression profiling in the experimental design. Then, excluding Sacheck et al. [5] datasets, ageing, unloading and two-months denervation seems to be representative of atrophy stimuli to which muscle react slower than fasting, denervation (from 1 to 14 days) and systemic states.

### Enrichments of putative transcription factor binding site (TFBS)

We have performed a search of putative TFBS separately for up or down regulated genes of each datasets. Table 5 lists the TFBS families that are enriched in the sequences of genes whose expression is altered in different types of muscle atrophy. As expected, the binding site of some known TF that contribute to skeletal muscle differentiation and gene expression (Myf, Mef2A, Sp1, SRF) have been detected as enriched in most of the atrophy datasets. Instead, some enriched TFBS correspond to transcription factors whose role in muscle atrophy was not yet evidenced. For example, DeltaEF1/ZEB1 is a transcriptional activator that in smooth muscle is directed at least in part toward mesenchymal genes such as collagens, smooth muscle actin and myosin, vimentin, and genes in the vitamin D signalling pathway, which is important in mesenchymal differentiation [39]. Recently, Chen et al. [40] demonstrate a cooperation between FoxO and deltaEF1 in activating growth suppressive genes in B lymphocytes. FoxO plays a central role in the development of muscle atrophy. Sandri et al. [16] in fact, showed that FoxO transcription factor induces the atrophy-related ubiquitin ligase atrogin-1 and MURF-1, and that their activation in skeletal muscle is sufficient to induce marked atrophy. Interestingly, several TFBS identified as enriched in the upstream/downstream regions of the differentially expressed genes, are recognized by TF whose mRNA results in turn differentially expressed. This is the case of MAX (up-regulated), MYC (down-regulated), and MEF2A (up-regulated). Further description and discussion of the results of TFBS search in atrophy datasets follow in the next section.

**Table 5:** Over represented transcription factor binding sites shared by differentially expressed genes across the different atrophic states.

**Up or down regulated genes**	**TFBS**	**Denervation**	**Fasting**	**Ageing**	**Diseases**	**Unloading**	**tot**
**-**	**SP1**	X		X	X	X	**4**
**-**	**MZF1_1–4**	X		X	X	X	**4**
**-**	**Myf**	X	X		X		**3**
**-**	**Roaz**		X	X	X		**3**
**-**	**deltaEF1**	X		X	X		**3**
**-**	**HAND1-TCF3**			X	X		**2**
**-**	**HNF4**		X	X			**2**
**-**	**MAX**	X			X		**2**
**-**	**MEF2A**	X	X				**2**
**-**	**NHLH1**	X			X		**2**
**-**	**PPARG**		X		X		**2**
**-**	**REL**			X		X	**2**
**-**	**RELA**			X		X	**2**
**-**	**SRF**		X			X	**2**
**-**	**TEAD**	X				X	**2**
**-**	**ZNF42_5–13**			X		X	**2**
**-**	**Arnt-Ahr**			X			**1**
**-**	**ELK4**		X				**1**
**-**	**Hox11-CTF1**		X				**1**
**-**	**MYC-MAX**	X					**1**
**-**	**Myb**				X		**1**
**-**	**NF-kappaB**					X	**1**
**-**	**NFKB1**	X					**1**
**-**	**NR2F1**		X				**1**
**-**	**RREB1**		X				**1**
**-**	**Spz1**					X	**1**
**-**	**TCF1**					X	**1**

**+**	**NHLH1**	X	X			X	**3**
**+**	**Staf**		X	X	X		**3**
**+**	**TEAD**	X	X	X			**3**
**+**	**ZNF42_1–4**	X	X			X	**3**
**+**	**Ar**		X		X		**2**
**+**	**E2F1**		X			X	**2**
**+**	**ELK4**		X		X		**2**
**+**	**MAX**		X		X		**2**
**+**	**Pax4**			X	X		**2**
**+**	**RREB1**			X	X		**2**
**+**	**SP1**	X				X	**2**
**+**	**deltaEF1**	X				X	**2**
**+**	**Arnt-Ahr**					X	**1**
**+**	**Bapx1**			X			**1**
**+**	**CREB1**		X				**1**
**+**	**ELK1**		X				**1**
**+**	**ESR1**			X			**1**
**+**	**FOXD1**				X		**1**
**+**	**FOXF2**				X		**1**
**+**	**Foxq1**				X		**1**
**+**	**HLF**			X			**1**
**+**	**Hox11-CTF1**	X					**1**
**+**	**IRF2**	X					**1**
**+**	**MEF2A**			X			**1**
**+**	**MYC-MAX**	X					**1**
**+**	**Myb**	X					**1**
**+**	**NFIL3**			X			**1**
**+**	**NFKB1**					X	**1**
**+**	**RELA**					X	**1**
**+**	**RORA1**					X	**1**
**+**	**RXR-VDR**				X		**1**
**+**	**SRF**			X			**1**
**+**	**TP53**	X					**1**
**+**	**ZNF42_5–13**					X	**1**

X indicates if the specific TFBS category is significantly enriched in that specific study. (-) TFBS enriched in the up/downstream regions of down-regulated genes; (+) TFBS enriched in the up/downstream regions of up-regulated genes

### Molecular networks

**Figure 3:**
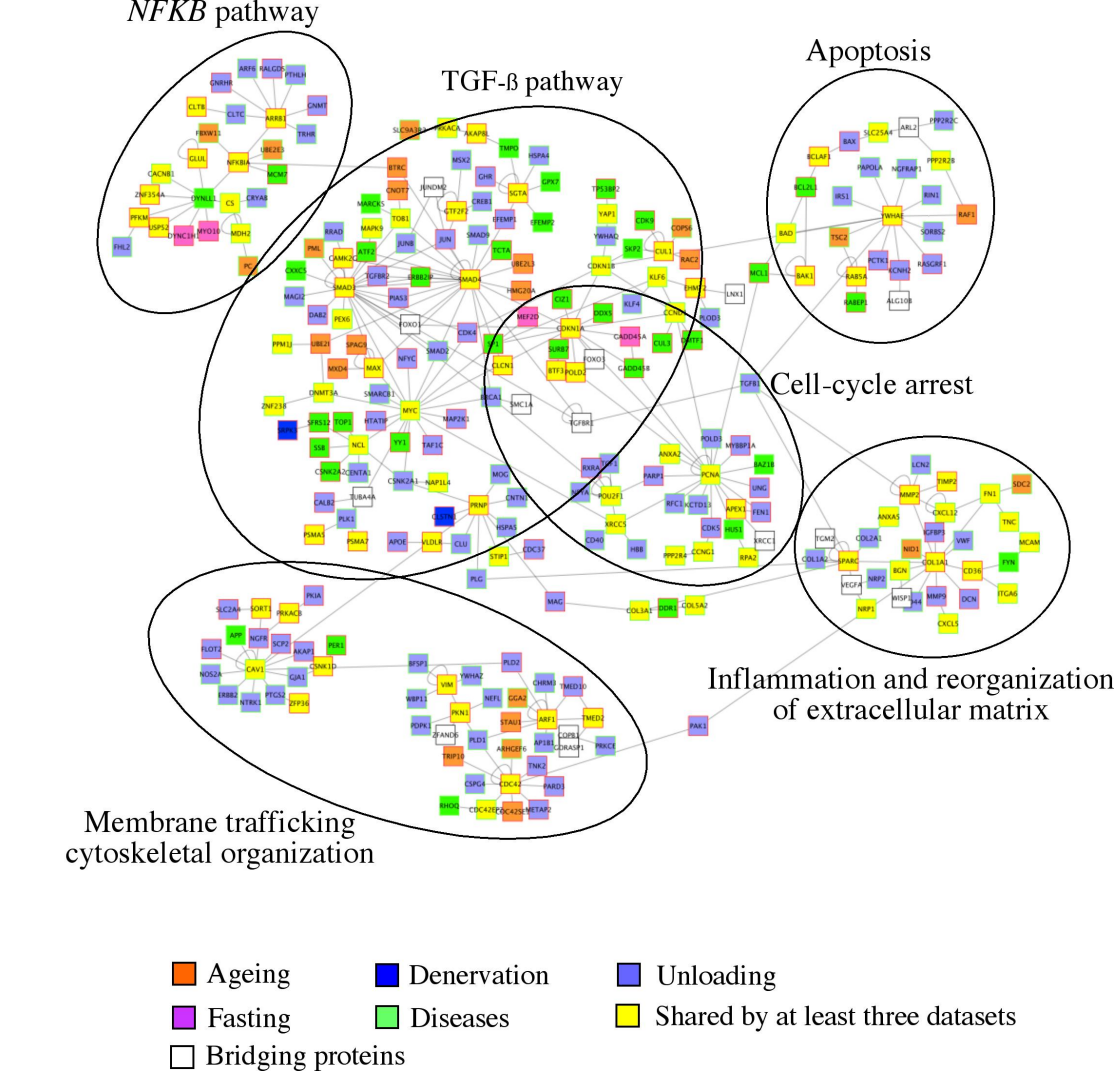
**Atrophy molecular network**. The whole molecular network was constructed through the integration of single networks computed from different atrophy expression datasets (denervation, unloading, fasting, diseases, ageing). Gene/protein nodes are represented by squares with identification symbols. Squares with red borders indicate up regulated nodes, whereas green borders indicate down regulated nodes. Colour of the symbol area identifies the expression dataset in which the corresponding gene was calculated as differentially expressed. Selection of network areas (black oval contours) has been performed focusing on hub genes/proteins. The whole network from which this zoomed network has been obtained is available in Additional file 1.

We constructed a general atrophy network, generated by the combination of dataset-specific networks, integrating expression levels and molecular interaction information. Under the assumption of linear relationship between transcript and protein levels, differential gene expression could give a clue of protein deregulation and of the alteration of signalling cascades to which they belong. In the network, nodes represent proteins whose level could be altered as a consequence of the muscle atrophic process, and edges represent functional interactions between nodes. Additional file 1 shows the complete network. The usual approach for functional interpretation of such huge network is based on network decomposition and extrapolation of most significant interactions. Hub proteins tend to be master genes responsible not only of the stability of the entire network but also of sub-networks. They represent key regulatory elements important to understand pathways involved in a physiological or pathological event. Therefore, we choose to select that proteins and specific module networks to: i) highlighting relevant interaction network ii) reducing network noise and iii) finding cluster representing protein complexes parts of key pathways. We select highly connected nodes (hub) and their nearest edges producing an highly-connected sub-network reported in Figure 3.

The molecular network is characterized by few highly connected nodes (SMAD3, SMAD4, MYC, CDKN1A, PCNA, CAV1, COL1A1, YWHAE, NFKBIA, ARF1, CDC42) most of which are present in more than 3 datasets (yellow nodes). Molecular network can be divided into regions representative of different cellular mechanisms: a) the TGF-β pathways that appears as the core pathway of the general atrophy network, b) the NFKB pathway and its correlated responses, c) the negative regulation of cell cycle, d) the response to apoptosis and inflammation.

#### TGF-β pathway

The molecular network assigns a central position to the up-regulation of SMAD3/4 and CDKN1A/p21 and to the down-regulation of MYC. Smad3 in fact, can mediate transcriptional repression of the growth-promoting gene MYC [41]. A complex containing Smad3, the transcription factors E2F4/5 (that recognizes the E2F1 site which is also over-represented in the sequence of genes of the datasets), DP1, and the co-repressor p107 is situated in the cytoplasm. In response to TGF-β, this complex moves into the nucleus and associates with Smad4, recognizing a composite Smad-E2F site on MYC flanking region for repression [41]. One of the paradoxes encountered by investigators studying Myc function is the observation that both Myc over and underexpression results in apoptosis. The link between Myc deficiency and apoptosis is less clear, but may also be mediated through a mitochondrial pathway [42]. SMAD complexes also could induce the transcriptional activation of genes encoding CDKN2B and p21/CDKN1A. This latter protein is upregulated in atrophic conditions and plays an important role in the inhibition of cell cycle progression [43]. Seoane et al. [44] identified *FOXO *proteins as key partners of Smad3 and Smad4 in the TGF-β-dependent generation of a CDKN1A/p21 activation complex. FoxO factors are under the negative control of the phosphatidyl insositol 3-kinase (PI3K) growth-promoting pathway [45]. In response to mitogenic signals, PI3K activates Akt, a protein kinase that phosphorylates FOXO, barring them from the nucleus and thus from target genes [46]. The present identification of FOXO factors as Smad partners in CDKN1A/p21 activation provides a link between the TGF-β/Smad and PI3K/AKT pathways and suggests a broader role for FoxO proteins as signal transducers. This link is particularly important considering that there are accumulating evidences that the PI3K/AKT pathway, a crucial intracellular signalling mechanism underlying muscle hypertrophy [47], prevents the induction of the two muscle-specific ubiquitin-ligases, atrogin-1 and Murf-1 in several models of muscle wasting [4,5]. Moreover, the mechanism for this prevention involves Akt-mediated inhibition of the FoxO family of transcription factors [16,17]. CDKN1A/p21 can associate to the proliferating cell nuclear antigen (PCNA), an auxiliary factor for delta and epsilon DNA polymerases. Cayrol et al. [48] demonstrated that the activation of CDKN1A/p21 may reduce cell cycle progression by inhibition of PCNA function resulting in cell cycle arrest both at G1 and G2. The consequence of this arrest could be that muscle cells undergo apoptosis (see Figure 3) or differentiation. In fact, Shen et al. (2006) [49] proposed the involvement of CDKN1A/p21 in the survival of muscle satellite cells. These cells are fundamental for the recovery of the tone when muscle tissue recoveries from an atrophy status, so the activity of CDKN1A/p21 is fundamental in this context.

#### Apoptosis, membrane trafficking and cytoskeletal organization

Cell cycle arrest in muscle cells can lead to apoptosis or differentiation. The apoptosis area of the network includes some members of the BCL2 family (such as BAD, BCL2L1, BAX and BCLAF1) well known to be involved apoptosis, as well as RAF1 and YWHAE proteins. This last gene product belongs to the highly conserved 14-3-3 family of proteins whose isoforms are associated with several intracellular signalling molecules in the regulation of various cellular functions, including cell cycle control, proliferation, transformation, and death by apoptosis.

On the other hand, differentiation and fusion of muscle cells into multinucleated myotubes is accompanied by a dramatic reorganization of the Golgi complex [50]. Schubert et al. [51] shows that skeletal muscle differentiation involves CAV-1 and CAV-2 genes down-regulation (evidenced also in this network). Many membrane-bound organelles, including endoplasmic reticulum (ER) and mitochondria, remain intact during mitosis. However the Golgi apparatus, which functions at the crossroads of many membrane trafficking pathways within cells [52], reversibly disassembles [53]. ARF1, a protein involved in the vesicular trafficking through Golgi, is also involved in myoblast differentiation during myotubes formation. In fact, it has been shown that myoblast expressing a mutant form of ARF1 fail to undergo differentiation and fusion [16]. Altan-Bonnet et al. [54] suggested that the inactivation of ARF1 early in mitosis could provide the release of a variety of proteins (whose functions are necessary for DNA replication, chromosome condensation, segregation, and cytokinesis) into the cytoplasm in a timely way so that they can carry out their respective mitosis-related activities. In fact, the blocking of ARF1 inactivation prevented membrane dissociation of many peripheral Golgi proteins and impaired two key events of mitosis, chromosome segregation and cytokinesis. ARF1 was found up-regulated in association to CDC42, a Rho protein whose activation is sufficient to promote cellular senescence such as muscle-ageing phenotype with muscle mass reduction. Furthermore, Takano et al. [55] suggested that Rho proteins play a critical role in muscle differentiation in relation to motility, shape and control of the actin cytoskeleton, possibly by regulating the expression of myogenin and MEF2 genes. In addition, the Rho and Cdc42 GTPases have also been implicated in TGF-β signal transduction.

#### Inflammatory response and reorganization of the extracellular matrix

Extracellular matrix (ECM) of connective tissues is important for force transmission and tissue structure maintenance in tendons, ligaments, bone and muscle. Several genes codifying ECM proteins (COL1A1, MMP2, MMP9, FN1, TIMP2) and implicated in its reorganization as well as in inflammatory processes have been identified in the atrophy networks. These data fit with the knowledge that muscle remodelling in consequence to atrophy includes an increased turnover of several ECM molecules.

### Short-term muscle response to atrophy induction

We have studied the presence of TFBS in the flanking regions of genes that are differentially expressed in at least three of the datasets pertaining to muscle short-term response to atrophy (less than 14 days after atrophy induction). The result of this analysis is reported in Table 6. A TFBS that appears to be over-represented is that recognized by MAX transcription factor. MAX gene is over-expressed in the same datasets, suggesting that these putative binding sites may be active.

**Table 6:** Transcription binding sites enriched in the flanking regions of genes that were calculated as differentially expressed in all 4 expression datasets of short-term muscle response to atrophy.

**TF**	**Target TFBS hits**	**Z-score**	**Fisher score**
**Hox11-CTF1**	17	15.97	5.031e-04

**TEAD**	65	14.69	1.232e-03

**MAX**	123	13.15	9.861e-04

**RREB1**	14	12.4	4.909e-03

**SP1**	315	10.86	1.786e-03

**Myf**	167	10.62	9.581e-02

**SRF**	13	10.61	1.351e-02

**ZNF42_1–4**	1287	10.13	4.823e-02

**NHLH1**	71	7.984	6.462e-03

**NR3C1**	31	7.805	6.044e-03

**Figure 4:**
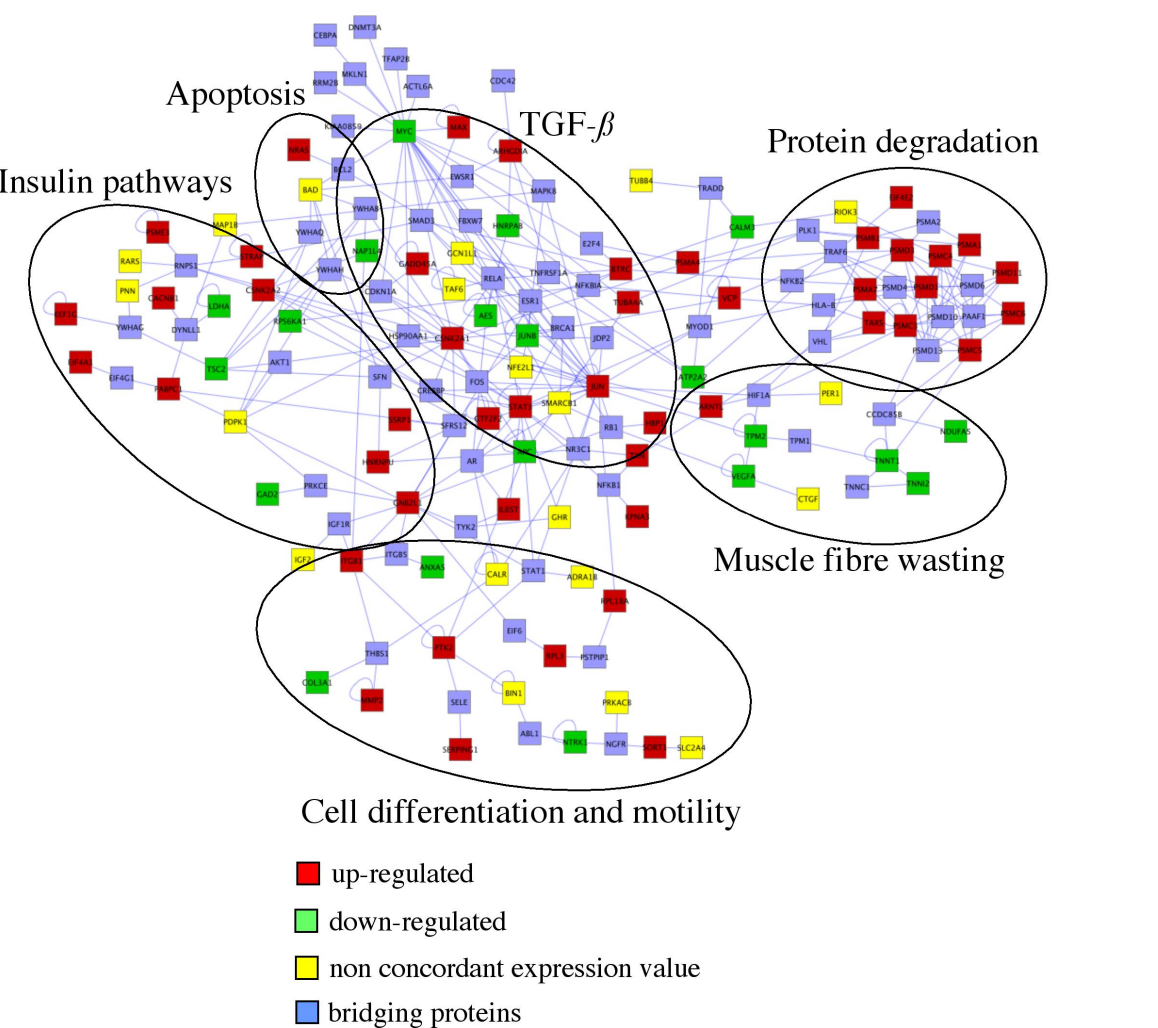
**Network of short-term muscle response to atrophy**. This molecular network has been constructed through the integration of single networks derived from expression datasets pertaining to muscles before 14 days from atrophy induction, and focusing on the hub genes/proteins. The complete network from which this zoomed network has been obtained is available in Additional file 2.

With the same approach described above, we have constructed a molecular network using datasets describing the short-term response to atrophy induction. Additional file 2 reports the complete network and Figure 4 shows a selection of the highly connected nodes. The goal of the analysis was the identification of pathways responsible of the initial phase of muscle remodelling. Interestingly, we found that a large area of the network contains genes related to proteasome and catabolism, suggesting that protein degradation is an early muscle reaction probably inhibited or balanced in long-term atrophy. Some muscle specific proteins, such as TNNT1, TNNI2 and some proteins related to energy production (NDUFA5, ATP2A2) with ARNTL, VEGFA and TPM2 seem to be involved in the process of muscle wasting. Few genes related to apoptosis (BAD and NRAS) are connected to a larger group of genes involved in the insulin signalling pathways (TSC2, RPS6KA1, CSNK2A2). The insulin receptor and insulin-like growth factor 1 receptor (IGF-1R), when activated by their ligands, control metabolism, cell survival, and proliferation in a variety of tissues, muscle included. Regulation of their activity is still under strong investigation. The over expression of GNB2L1/RACK1 inhibits phosphorylation of AKT induced by IGF-1. This result suggests that GNB2L1/RACK1 has a particular role in regulating Akt activation and cell survival [56]. CSNK2A1/CKII is a protein kinase that phosphorylates *in vivo *and *in vitro *a variety of transcription factors, either gene expression activators, such as Myc, c-Jun, Sp1, or repressors. Phosphorylation can result in either positive or negative modulation of their activity [57].

The transcription factors MYC and MAX represent hub proteins also in the specific network that describes the molecular interactions involved in the early steps of muscle atrophy and their central role in the general biological processes related to atrophy is therefore reinforced. SMAD, that has been identified as one of the hub proteins in the general atrophy network (Figure 3), in this network seems to be replaced by JUN, JUNB and STAT3 (Figure 4). JUN is a transcriptional activator associated with rapid cellular growth and was found down-regulated in the expression studies of muscle atrophy performed by Lecker et al. [4] and Sacheck et al. [5]. It has been shown that SMAD3 and 4 proteins interact with AP-1 family of transcription factors among which are included some members of the Jun family like JUN, JUNB and JUND [58]. Genes of the c-Jun family together with Myc are called immediate early genes (IEGs), that are genes activated transiently and rapidly in response to a wide variety of cellular stimuli [59]. These genes play important roles in signal transduction and transcriptional regulation in normal cells coupling extracellular stimuli to changes in cellular phenotype. Thus, many of IEGs encode TFs that are rapidly induced in response to a wide list of physiological and pathological conditions. Therefore, this seems to be in good agreement with our identification of c-JUN family as hub proteins in the short-term muscle response to atrophy. In fact, impairment of the of the AP-1/c-jun signalling cascade by c-JUN down regulation in vivo, is able to partially counteract muscle mass loss in a rat model for cachexia [60].

## Conclusion

In this work we applied a meta-analysis approach in order to verify the similarities in the molecular pathways underlying diverse skeletal muscle atrophies induced by different stimuli. Transcriptome data has been integrated with molecular interaction data, in order to construct a general network descriptive of the molecular processes involved in the establishment and maintenance of muscle atrophy.

We found a general down-regulation of genes involved in energy production and carbohydrate metabolism and, in contrast, up-regulation of genes with role in protein degradation and catabolism. This result was expected, since muscle wasting that accompanies atrophy is caused by imbalance between protein synthesis and protein degradation. According to the gene networks, it appears that the short-term response of muscle to atrophy is involving slightly different functional classes and members than the long-term response. The general molecular network that we have constructed from the analysis of specific networks obtained for different types of muscle atrophy gives a complete overview of the interconnected molecular pathways that have been implicated in muscle degeneration caused by atrophy. The analysis of the network has revealed some key factors (hub genes/proteins) that may have central roles in molecular processes associated to atrophy.

As most of the biological networks, the topology of the atrophy network we have assembled has a scale-free structure characterised by hundreds of nodes with only few of them showing a high number of connections. These hub nodes point to six different pathways that could be therefore considered as central for muscle atrophy process; these are the TGF-β pathway, apoptosis, membrane trafficking/cytoskeleton organization, NFKB pathway, inflammation and reorganization of the extracellular matrix.

The molecular pathway for protein degradation is present only in the network representing muscle short-term response to atrophy. This result supports the hypothesis that protein degradation is an early response to the stimulus inducing atrophy that is subsequently hidden probably by biological processes of muscle adaptation to atrophy. The central role in the atrophy network is assigned by our analysis to the TGF-β pathway with SMAD3/4, MYC, MAX and CDKN1A in the general networks that are substituted by JUN, MYC, GNB2L1/RACK1 in the short-term network.

Considerable progresses have been made in the identification of cellular signals regulating skeletal muscle atrophy, but our knowledge about the molecular mechanisms underlying atrophy is still partial. To date, there are no pharmacological treatments for disuse atrophy and electrical stimulation to maintain muscle tone is still the primary method used to inhibit muscle loss during extended periods of inactivity. For muscle diseases, sodium butyrate has been used to ameliorate a symptoms of SMA [61] and the only established treatment for muscular dystrophy is the use of steroids such as prednisone and deflazacourt [62-64]. These treatments however can only slightly counteract the important loss of muscle tissue associated to muscular dystrophies, while are producing significant side effects. Furthermore, the gene manipulation experiments that have been successful at maintaining muscle mass [65,66] have not been yet translated into therapeutic strategies. In summary, despite the progresses that have been made in identifying key elements associated with skeletal muscle changes during growth [11,21,67], apoptosis [68,69], and protein degradation [70-72], there has been limited success in attenuating the effects of atrophy on muscle tissues associated to pathophysiological processes. Genomic studies at transcriptional and proteomic level are revealing that the dissection of signalling pathways has not completely defined all the required elements necessary to maintain muscle mass neither identified whether a common "atrophy program" is activated by the various perturbations that produce the atrophic response. A major challenge now is to complete the description of the pathways responsible for the multiple intracellular signalling cascades in atrophic skeletal muscle and their interconnections, trying to identify some key factors that could be further studied as potential target for therapeutic purpose. In this perspective, we think that our approach has given a good contribution: in fact we were able to identify some proteins and transcription factors, such as SMAD3/4, GNB2L1/RACK1, MYC, MAX and JUN whose functions have been studied extensively in tumours [73-75] and in some atrophy models [59,60,76]. We suggest that these proteins could play important roles in the response of muscle to atrophy, and that further investigations on their role in skeletal muscle will greatly contribute to the comprehension of this complex process.

## Methods

### Data Collection

Expression datasets selected in this study are publicly available at Gene Expression Omnibus (GEO) database at NCBI or freely accessible to Author's web site. Only datasets whose raw data were publicly available have been considered in this study. Datasets analyzed were produced by the following Authors: i) Kostrominova et al. [37], concerning long-term denervation in rat, using membrane arrays (GSE1741), ii) Raffaello et al. [34] concerning molecular alterations at one, three, seven, and fourteen days after denervation in mouse (GSE1893), iii) Sacheck et al. [5], describing expression changes in disuse atrophy induced by denervation or spinal cord isolation in rat (available at ), iv) Welle et al. [32], a study of gene expression changes related to skeletal muscle ageing in human by oligonucleotyde array (GSE362), v) Stevenson et al. [33], an investigation on molecular alterations in skeletal muscle due to muscle inactivity in rats (generously provided by the Authors), vi) Jagoe et al. [20], who used cDNA microarrays to define transcriptional changes triggering muscle atrophy and energy conservation due to food deprivation in mice, vii) Lecker et al. [4], who identified a common set of transcriptional adaptations underlying the loss of muscle mass caused by cancer cachexia, renal failure and diabetes. These two last datasets are available at . We download and then analysed only microarray experiments obtained through hybridization of rat muscle mRNAs to mouse array platforms. A detailed description of datasets included in the meta-analysis is available in Table 1.

### Statistical Analysis

#### Normalization

Expression quantification for Affymetrix CEL files [32,33] has been done using EntrezGene Custom CDF (corresponding to 11,991 transcript for HG-U133A and 4,029 for the U34A) annotation files proposed by Dai et al. [77], then rma algorithms [78] have been performed with WGAS web tool [79] for normalizing data. Raw data derived by two-colour cDNA microarray have been normalized using lowess algorithm [80] with MIDAW web tool [81].

#### Identification of differentially expressed genes

Permutational t-test has been performed to identify differentially expressed genes in all those studies with the case vs control experimental design. P-values and Q-values (false discovery rate, FDR) [82] have been used as ranking. Q-values for each gene has been defined as: Q = (p*n)/*i*, where *p *is the *p-value *of the gene, *n *the total number of genes and *i *is the number of genes at or better than *p*. In the case of time-course experimental design, permutational two-way ANOVA has been performed considering in the model treatment effect and (time × treatment) interaction. In this way we identified genes differentially expressed between normal and atrophic samples across and within time points.

Unfortunately not all the datasets contain sufficient numbers of biological replicates as required for powerful inference. In particular, Lecker et al. [4] and Sacheck et al. [5] performed less than 3 replicates respectively for each type of samples analyzed (uremia, diabetes and tumour) and for each disuse-induced atrophy (denervation or spinal cord) they preferred the use of pooled samples. Fold change cut-off, usually applied to microarray data in case of insufficient number of replicates, leads to large number of false positive. Therefore, to settle this problem, we decided to combine muscle wasting experiments and atrophy induced by denervation plus by spinal cord damage respectively in two separate datasets. Then, we constructed an expression matrix where the number of rows matches the number of genes represented in the microarray platform used by Lecker et al. [4] and where there are 5 columns as the number of experiments: 1 experiment for diabete, 2 experiments for tumor and 2 experiments for uremia. The same approach has been used for Sacheck et al. [5] dataset: the final expression matrix is composed by as many rows as the number of genes of the array used in Sacheck et al. [5] and 4 columns equal to the number of experiments: 2 experiments for 3 days denervation, 2 experiments for 3 days spinal cord. In this way, sample replicates increase and t-test approach can be applied. Statistical test for the identification of differentially expressed genes has been applied to these two final matrices. A FDR ≤ 0.1 has been used to choose significant gene lists. Statistical inference has been performed with R software  with DAAG package.

#### Meta-analysis and gene list comparison

Given the different model organisms used in expression studies (*Homo Sapiens*, *Mus Musculus *and *Rattus Norvegicus*) we used HomoloGene database to identify homologous genes in order to match different studies. *Homo Sapiens *has been used as reference organism. After homologous conversion of all the lists of differentially expressed genes, we followed an approach highly similar to those proposed by Rhodes and colleagues [83] to identify a significant *meta-signature*, defined as a selected set of genes common to *j *of the *S *total number of datasets. The number *j *is defined through a permutational approach. The idea is to compare the observed number of significant genes shared by at least *j *studies (observed gene enrichment) with the number of significant genes shared by at least *j *studies obtained by chance (random gene enrichment). Permutational steps are as follows: i) Q values of each dataset are randomly permutated so that genes in each signature (list of differentially expressed genes) change at random, but the number of genes in each signature remains the same, ii) the number of genes differentially expressed common to at least *j *datasets are calculated for *j *ranging from 2 to the total number of datasets, iii) step i) and ii) are repeated 1000 times, iv) average and empirical confidence intervals (at confidence level 95%) of the number of random gene enrichment for each *j *(across the 1000 simulations) are calculated. Then, we compared the observed number of genes shared by at least *j *studies with the confidence interval obtained through the permutational approach and choose that *js *showing a significant difference between observed and random number of gene enrichment. Finally among these *j*s we select the minimum *j *such that the ratio between the expected and observed number of gene shared is less than 10%.

### Functional classification and transcription factor binding site search for differentially expressed genes

Functional classification of gene lists has been performed for each dataset. Differentially expressed gene has been associated to one or more Gene Ontology (GO) categories and KEGG metabolic pathways using BABELOMICS tool [84]. Class enrichment (with respect to the entire platform) has been calculated with the hypergeometric distribution (Fisher exact test). The hypergeometric distribution is used to obtain the chance probability of observing the number of genes from a particular GO/KEGG category among the selected differentially expressed genes. The probability *P *of observing at least *k *genes of a functional category within a group of *n *genes is given by:

$$P=\sum_{i=k}^{n}\frac{\left(\begin{array}{c} f\\ i \end{array}\right)\left(\begin{array}{c} g-f\\ n-i \end{array}\right)}{\left(\begin{array}{c} g\\ n \end{array}\right)}$$

where *f *is the total number of genes with the same GO class (in the microarray platform) and *g *is the total number of genes within our platform. Then, the lists of the significantly enriched GO categories and KEGG pathways (FDR < = 0.1) for each study have been compared.

Over-represented putative transcription factor binding sites have been detected for the lists of differentially expressed genes with oPOSSUM web tool [85]. The default parameters suggested by the Authors have been used to find TFBSs in the genomic flanking regions upstream and downstream the sequences of co-expressed genes. Two statistical measures (Z-score and Fisher exact one-tail probability) were calculated to determine which TFBS were significantly over-represented in the examined flanking regions. Z-score > 6 and Fisher *p-value *< 0.01 were used as significant cut-off thresholds.

### Networks constructions

Gene expression levels and molecular interaction information have been integrated in order to construct a molecular network of atrophy. Protein interaction file has been downloaded from the NCBI ftp site. NCBI integrates protein interaction information from three different source databases: BIND (Biomolecular Interaction Network Database, ) [86], BioGRID (Biological General Repository for Interaction Datasets, ) [87], HPRD (Human Protein Reference Database, ) [88]. From the entire file we selected only those interactions where one or both of the two partner proteins were produced by genes differentially expressed in our datasets. Cytoscape software [89] has been adopted to visually integrate molecular information. Nodes of the networks correspond to genes differentially expressed in datasets while edges correspond to protein interactions. BINGO plug-in [90] has been used to assess over representation of gene ontology categories in the considered biological networks.

## Authors' contributions

EC performed all the statistical and bioinformatic analysis (normalization, statistical test, metabolic pathways, biological processes and TFBS enrichment, network construction). SC, AR, and PL participated in the design of the study, revised the manuscript and participated in the investigation of the differentially expressed genes and interpretation of the results. CR and GL conceived and supervised the study, write the manuscript, coordinate the work and the interpretation of the results. All authors read and approved the final version of the manuscript.

## Supplementary Material

Additional file 1 **Complete atrophy molecular network.** The whole molecular network was constructed through the integration of single networks computed from different atrophy expression datasets (denervation, unloading, fasting, diseases, ageing). Gene/protein nodes are represented by squares with identification symbols. Squares with red borders indicate up regulated nodes, whereas green borders indicate down regulated nodes. Colour of the symbol area identifies the expression dataset in which the corresponding gene was calculated as differentially expressed. Click here for file

Additional file 2 **Complete network of short-term muscle response to atrophy.** This molecular network has been constructed through the integration of single networks derived from expression datasets pertaining to muscles before 14 days from atrophy induction, and focusing on the hub genes/proteins. Click here for file
