# Supplementary figures and images for: Meta-analysis of expression signatures of muscle atrophy: gene interaction networks in early and late stages
Source: BMC Genomics. 2008 Dec 23;9:630. doi: 10.1186/1471-2164-9-630 (PMC2642825; doi:10.1186/1471-2164-9-630)

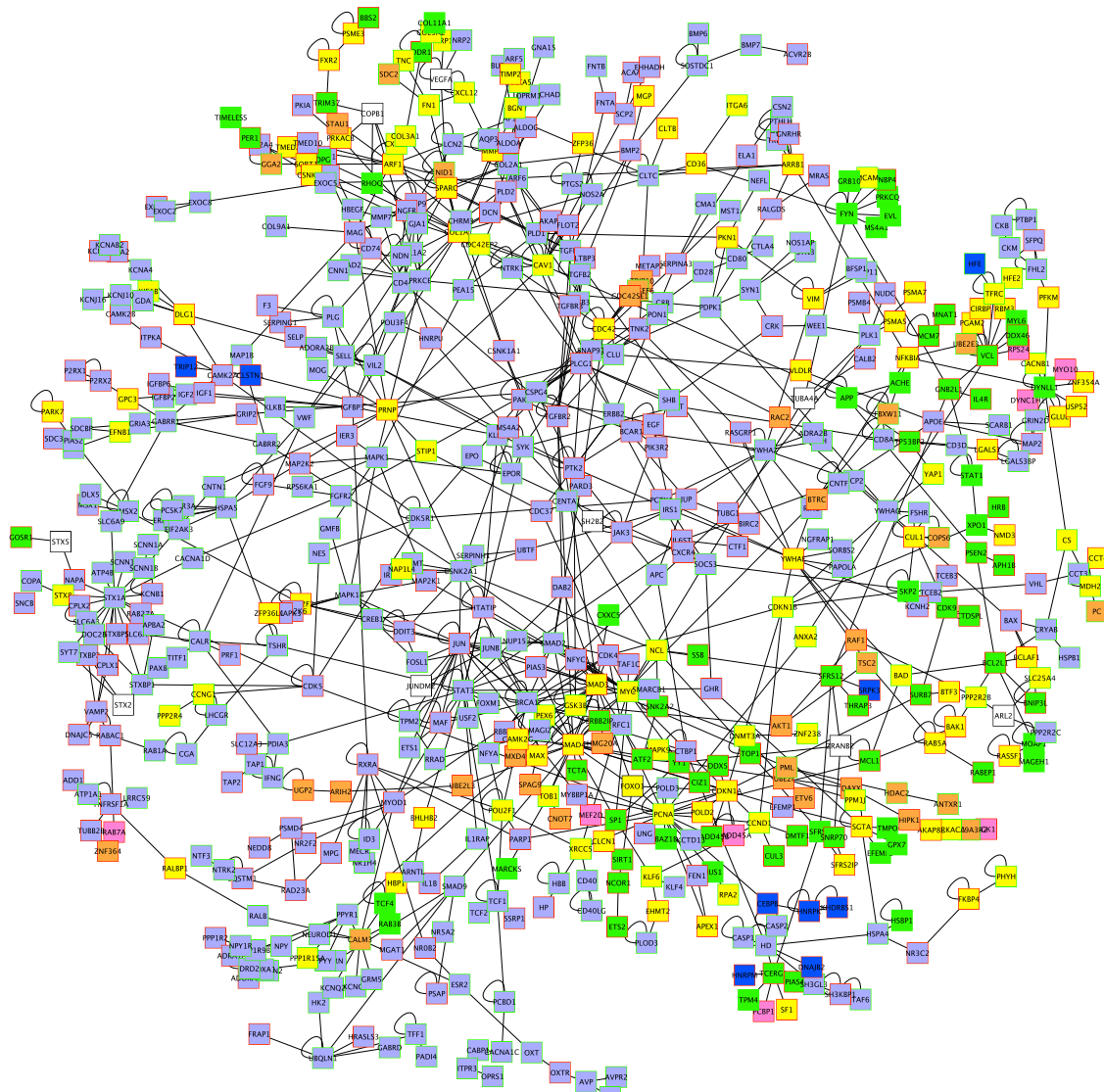

Supplement: Additional file 1 — Complete atrophy molecular network. The whole molecular network was constructed through the integration of single networks computed from different atrophy expression datasets (denervation, unloading, fasting, diseases, ageing). Gene/protein nodes are represented by squares with identification symbols. Squares with red borders indicate up regulated nodes, whereas green borders indicate down regulated nodes. Colour of the symbol area identifies the expression dataset in which the corresponding gene was calculated as differentially expressed. [file 1471-2164-9-630-S1.pdf]

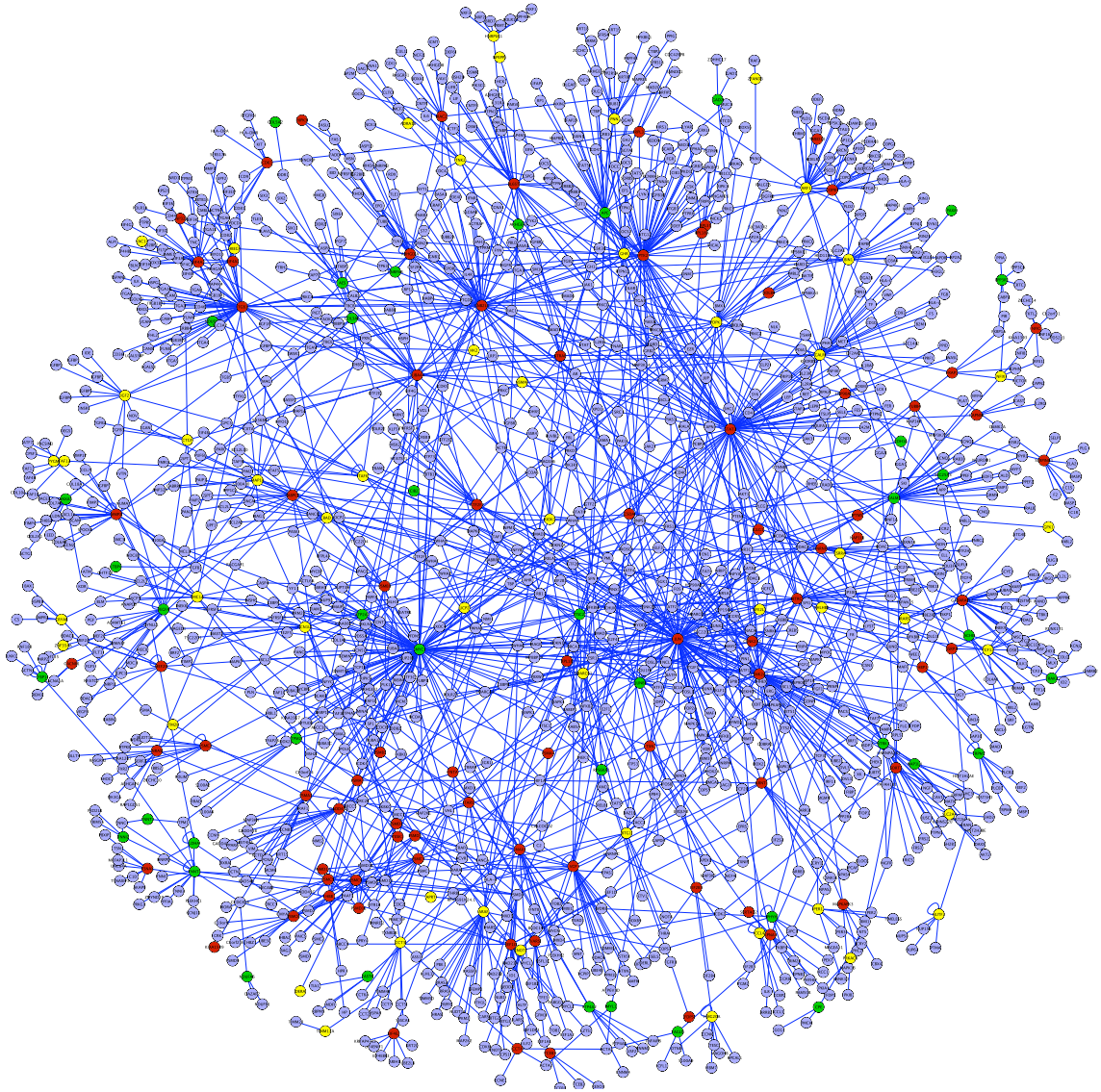

Supplement: Additional file 2 — Complete network of short-term muscle response to atrophy. This molecular network has been constructed through the integration of single networks derived from expression datasets pertaining to muscles before 14 days from atrophy induction, and focusing on the hub genes/proteins. [file 1471-2164-9-630-S2.pdf]
